# Editorial: Empowering cancer care: the power of nutrition and fitness from prevention to recovery

**DOI:** 10.3389/fnut.2026.1938940

**Published:** 2026-08-14

**Authors:** Irene Lidoriki, Maximos Frountzas, Panagiotis Sakarellos, Dimitrios Schizas

**Affiliations:** 1 Harvard T. H. Chan School of Public Health Department of Environmental Health, Boston, MA, United States; 2 Department of Occupational Medicine, Cambridge Health Alliance, Cambridge, MA, United States; 3 Department of Colorectal Surgery, The Royal Marsden NHS Foundation Trust, London, United Kingdom; 4 First Department of Surgery, Ethniko kai Kapodistriako Panepistemio Athenon Geniko Nosokomeio Athenon Laiko, Athens, Greece

**Keywords:** cancer, dietary habits, dietary patterns, fitness, metabolism, nutrition, nutritional interventions, physical activity

Cancer constitutes one of the leading global health challenges, demanding a sophisticated, multidimensional approach across prevention, acute treatment, and survivorship. Growing evidence highlights the crucial role of nutrition and physical fitness in reducing cancer risk, enhancing treatment efficacy, and improving clinical outcomes. Today, as treatment modalities advance and survivorship expands, the focus has broadened beyond pure survival to encompass the preservation of functional capacity, metabolic stability, and overall quality of life. Indeed, quality of life has emerged as a paramount outcome in contemporary oncology, particularly as early detection and therapeutic innovations increasingly transform cancer into a manageable chronic disease. Mental health is also linked to physical wellbeing; yet, psychological support and lifestyle modifications remain vastly underutilized in standard cancer care. Understanding and optimizing the intricate interplay between diet, exercise, and psychological resilience—and how these factors modulate biological cancer pathways—is essential for empowering the general population, patients, and healthcare professionals alike.

This Research Topic, “*Empowering cancer care: the power of nutrition and fitness from prevention to recovery*,” brings together 20 pivotal contributions that bridge the gap between lifestyle habits, cancer risk, and survival. Spanning a diverse spectrum—from large-scale epidemiological data associating daily habits with malignancy risk, to precise pre-treatment clinical assessments, to the implementation of innovative interventions in both hospital and home-based environments—this Research Topic illuminates the profound impact of nutritional and physical care across the entire cancer continuum. Moving from molecular mechanisms to digital home-care platforms and trimodal prehabilitation, these articles highlight a critical truth: optimal modern oncology requires the timely and systematic integration of lifestyle interventions.

## Epidemiology, prevention, and predictive risk

The foundation of empowering cancer care begins before diagnosis. Large-scale epidemiological studies within this Research Topic demonstrate that dietary quality is a potent, modifiable risk factor in cancer prevention. Utilizing data from over 101,000 adults in the PLCO Cancer Screening Trial, Feng, Wen et al. demonstrated that adherence to a diet with high protein quality significantly reduces both the incidence and mortality of colorectal cancer (CRC). Expanding on this within the same massive cohort, the authors Feng, Xiang et al. further revealed that a high Macronutrient Quality Index (MQI)—accounting for the holistic quality of carbohydrates, fats, and proteins—yields a 22% lower incidence and a 38% lower mortality from CRC, with a remarkable 36% incidence reduction specifically for distal colon cancer. Globally, the systemic impact of dietary patterns remains substantial; Tian et al. utilized Global Burden of Disease (GBD) data from 204 countries over 30 years (1990–2021) to highlight that despite a 2.26% annual decline in age-standardized mortality, the burden of gastric cancer attributable to high-sodium diets remains a critical public health challenge, particularly in transitioning economies. Beyond specific nutrients, baseline body composition serves as a vital predictive tool. A systematic review of over 1.5 million participants by Liu, Fu et al. investigated the “A Body Shape Index” (ABSI), revealing that each one-standard-deviation increase in ABSI corresponds to an 8% increase in overall CRC risk, serving as a highly specific supplementary screening tool for central adiposity.

When transitioning from prevention to clinical treatment, baseline nutritional screening becomes a critical determinant of therapeutic tolerance. In a retrospective analysis of 142 acute myeloid leukemia patients, Guo et al. found that a pre-transplant Body Mass Index (BMI) of less than 24 kg/m^2^ acts as an independent risk factor for inferior overall survival (HR = 1.80). Interestingly, for high-risk patients, low BMI caused 3-year survival rates to plummet from 57.69% to just 20.87%. Similarly, Xu et al. successfully developed and validated a nomogram in 302 patients incorporating the Controlling Nutritional Status (CONUT) score, which outperformed existing tools (AUC 0.815 vs. 0.706) to precisely predict prolonged hematological toxicity following CAR-T cell therapy. To standardize how we measure these complex variables, Chen, Sun et al. developed a highly reliable 43-item predictive model in a 300-patient cohort based on the International Classification of Functioning, Disability, and Health (ICF), offering clinicians a robust tool to evaluate multidimensional nutrition-associated health levels.

To effectively intervene, we must understand the biological mechanisms linking physical decline to cancer progression. A prospective pilot study of 43 CRC patients by Perillo et al. revealed that preoperative malnutrition is directly associated with suppressed intratumoral T-cell function, increased neutrophil infiltration, and a distinct, dysbiotic tumor-associated microbiota. Complementing this, a comprehensive review by Liu, Yang et al. explored the diet-microbiome-immune axis in prostate cancer, illustrating how plant-based diets foster anti-inflammatory microbial profiles, whereas Western diets drive the dysbiosis and metabolite production that fuel tumorigenesis.

## Active interventions: multimodal care and prehabilitation

Remarkably, physical rehabilitation appears to exert similar microenvironmental influence. A narrative review by Chen, Zhang et al. synthesized compelling preclinical and clinical evidence that rehabilitation interventions (including exercise therapy, physical agent modalities, occupational therapy, and mental health support) do not merely improve strength, but actively impede cancer metastasis by modulating the immune system, reducing chronic inflammation, and altering the tumor microenvironment.

Sarcopenia has emerged as a significant prognostic factor in patients with cancer; therefore, interventions that improve this condition are of considerable clinical importance. A comprehensive meta-analysis by Zhao et al., encompassing 59 randomized controlled trials (RCTs), demonstrated that targeted interventions for sarcopenia significantly improve muscle mass and physical performance outcomes, including the 30-s sit-to-stand test, in oncology patients. Notably, multi-component interventions combining nutritional support and exercise were associated with the greatest efficacy.

One of the most debated topics in contemporary cancer care is the concept of prehabilitation. Its potential clinical value is highlighted by the study of Deng et al. who implemented a 4-week trimodal prehabilitation program in 97 patients with stage IV triple-negative breast cancer. Their intervention significantly improved patients' quality of life and reduced anxiety. Interestingly, it was also associated with longer progression-free survival compared with the control group (298 vs. 235 days).

However, intervention success relies heavily on patient engagement. A latent profile analysis of 570 oncology patients by Meng et al. identified that 14.6% of patients fell into a “High Fatigue–Low Nutritional Awareness” profile, highlighting the need for concurrent psychological and educational support during therapy.

## Survivorship: bridging the gap from hospital to home

As patients transition from acute care to survivorship, continuity of care becomes increasingly important. Modern oncology must extend beyond the hospital walls. Reflecting this shift, Wang et al. implemented a “Hospital-to-Home” (H2H) nutrition management model for 100 patients with gastrointestinal cancer undergoing chemotherapy. The intervention significantly increased serum albumin levels (+2.7 g/L) and CD4+ immune cell counts while reducing the incidence of adverse reactions from 20.0% to 4.0%.

Digital health technologies can further strengthen this continuum of care. Yao et al. developed a remote exercise management platform tailored for colorectal cancer (CRC) patients with intestinal stomas, achieving an excellent System Usability Scale score of 88.39 among 62 participants. Nevertheless, the human element remains central. Liao et al. qualitatively explored the experiences of 13 post-esophagectomy patients and 15 caregivers managing home enteral nutrition, highlighting the critical need for improved health literacy and stronger community-based support.

Physical and psychological rehabilitation are intrinsically linked in survivorship. For post-mastectomy patients, Elsherbini et al. demonstrated that integrating Kinesio-taping with standard chest physiotherapy significantly reduces visual analog pain scores (3.97 vs. 6.50) while enhancing forced vital capacity. Meanwhile, tracking 50 postoperative breast cancer patients, Fei et al. utilized path analysis to reveal that functional exercise compliance is a critical protective factor against the fear of cancer recurrence. Broadening the scope of “fitness” to encompass mental resilience, a meta-analysis of 47 RCTs involving 4,537 patients by Li et al. confirmed that mind-body exercises (like yoga and mindfulness) effectively alleviate anxiety, depression, and fatigue, while concurrently reducing systemic inflammatory markers like IL-6.

## Future frontiers

The culmination of this Research Topic reflects a broader evolution in oncological sciences. A comprehensive bibliometric analysis of 2,887 publications by Gao et al. maps this global shift perfectly: over the past two decades, research on frailty and nutrition in cancer has moved decisively from descriptive epidemiology toward precision, evidence-based, integrative frameworks combining clinical nutrition, geriatric oncology, and rehabilitation science.

Empowering cancer care requires recognizing nutrition and physical fitness not merely as modifiable risk factors, but as fundamental pillars of comprehensive oncological treatment. By embracing multimodal, personalized, and technology-enabled interventions from the time of diagnosis through long-term survivorship, we have the opportunity to meaningfully improve both the quality and the duration of our patients' lives.

